# Planning and Promoting events in Health Sciences Libraries: Success Stories and Best Practices (book review)

**DOI:** 10.29173/jchla29593

**Published:** 2021-12-01

**Authors:** Sarah Visintini

**Affiliations:** MLIS, Berkman Librarian, University of Ottawa Heart Institute Ottawa, ON, Canada

Gillum, S., Williams, N. **Planning and promoting events in health sciences libraries: Success stories and best practices**. 1st ed. Maryland; Rowman & Littlefield; 2021. Softcover: 153 p. 978-1-5381-3590-7. Price: USD $65.00. Available from: https://rowman.com/ISBN/9781538135907/Planning-and-Promoting-Events-in-Health-Sciences-Libraries-Success-Stories-and-Best-Practices

The title says it all: *Planning and promoting events in health sciences libraries* is a book intended as a practical guide for planning and implementing successful programs and events in the health sciences library context. The book is comprised of two parts. The first portion is instructional. Think – how to take stock of existing programming, resources, and user needs, as described in Chapter Two. The second half includes case studies that provide ideas, break down approaches used, and detail lessons learned. An example is the creation of a monthly crafting “Fun Lab” through the Hirsh Health Sciences Library at Tufts University in Boston described in Chapter Twelve.

The editors, Gillum and Williams, are both academic health sciences librarians at the University of Central Florida. Their impressive list of contributors consists of both librarians and library technicians in primarily academic health sciences libraries at various career stages in the United States. Of the twenty-three contributors to this book, two are hospital library workers, and one works in a special library.

To my knowledge, this is the only book on library programming that specifically addresses event planning in a health sciences library context. Other more general publications do exist, such as *Great Library Events: From Planning to Promotion to Evaluation, Planning Academic Library Orientations: Case studies from around the world*, and *Library programming made easy: A practical guide for librarians*. Having not had the pleasure of reading these other books, it would be unfair to compare them; however it’s probably safe to assume that not all of the examples or cases provided would be relevant to our discipline, as compared to those included in Gillum and Williams’ book. I found reading about these examples quite powerful, since it felt like I received permission and precedent to envision the kind of fun, light-hearted programming we typically associate with a public library, but targeted towards our unique populations and with the purpose of strengthening our ties to them. As Gillum and Williams press several times in the introduction, conception and design of this book: “ideas like these are not out of your reach or beyond the scope of the work you do as librarians.”

While clearly meant for health sciences library workers, it is evident from the examples given and the contributor affiliations that this book was primarily targeted to an audience from an academic setting. As an academic librarian embedded in a clinical environment, I found much of their content relatable and thoughtful, and still relatively applicable in a more budget-constrained setting. For example, contributors mentioned working solo just as often as putting on programs as a team, and while the approach to budgeting and finding funding for events was from the academic perspective, some of the strategies suggested could also be applied in a clinical library setting. Even if the budgets proposed in this book are outside the scope of what non-academic libraries could hope to gather for their events, I do think there are enough practical tips and idea generating examples of value to those readers. That said, librarians working exclusively with researchers or clinical staff at non-teaching institutions may find the second portion of the book less helpful, since they are for the most part targeted at student or student-mix populations.

This book would be a great read for library workers at any stage of their career who are new to programming, do programming along with a million other things and could use some optimization (this is me), or who are experienced in providing programming but are looking for ideas and approaches to shake things up. As a member of the middle, slightly frazzled group, I found it helpful not only because of the interesting case studies, but also for providing a cohesive framework to approach programming, since as a solo librarian event planning can often feel sporadic, short notice, and disorganized.

I found this to be a very easy read, with all the contributors managing to hit the same or similar approachable tones. The level of detail and images provided were great for giving the reader a sense of the events. I really appreciated the “lessons learned” sections of each of the case studies, as they did not shy away from highlighting their weaknesses or missteps. I also saw similarities to my own programming in some of the examples provided, so it was helpful to read a break down of what was not working, how they aimed to fix it, and think about how this might apply in my own context.

While I did find a lot of the content relevant to me, I would have liked to see a bit more about hospital and governmental health libraries, since they can both be constrained in unique ways. Similarly, I would have been interested to read about programming from relevant international jurisdictions, to help readers break out of the echo chambers we can sometimes create within our own national conferences and professional networks.

My final recommendation: I found this a worthwhile, approachable read. The first section of the book was helpful for me to rethink and re-organize my own practices, and I found myself inspired by a number of the case studies provided in the second section, despite differences in our target demographics and settings. Since reading this book I’ve already made some changes to how I send out communications in order to present more cohesive branding, and I plan on incorporating aspects of some of the case studies presented into my own outreach moving forward. Namely, I’m going to be on the lookout for a prize wheel!

## References

[1] Flaherty MG. Great library events: From planning to promotion to evaluation. United States: Rowman & Littlefield Publishers; 2021. Available from: https://rowman.com/ISBN/9781538137062/Great-Library-Events-From-Planning-to-Promotion-to-Evaluation

[2] Bailin K, Jahre B, Morris S. Planning academic library orientations: Case studies from around the world. Cambridge, MA: Chandos Publishing; 2018. https://www.elsevier.com/books/planning-academic-library-orientations/bailin/978-0-08-102171-2

[3] Demeter M, Holmes HK. Library programming made easy: A practical guide for librarians. Lanham, MA: Rowman & Littlefield Publishers; 2019. https://rowman.com/isbn/9781538117026

